# Influence of Pre-Analytical Factors on Thymus- and Activation-Regulated Chemokine Quantitation in Plasma

**DOI:** 10.5772/61749

**Published:** 2015-10-27

**Authors:** Xuemei Zhao, Liliana Delgado, Russell Weiner, Omar F. Laterza

**Affiliations:** 1 Translational Molecular Biomarkers, Merck Research Laboratories, Rahway, NJ, USA; 2 Translational Sciences, Biomarkers & Companion Diagnostics, Daiichi Sankyo, Inc., Edison, NJ, USA

**Keywords:** Pharmacodynamic biomarker, Pre-analytical factors, Platelet-free plasma, Platelet-containing plasma, Freeze-thaw, Thymus, Activation-regulated chemokine, Atopic dermatitis

## Abstract

Thymus- and activation-regulated chemokine (TARC) in serum/plasma associates with the disease activity of atopic dermatitis (AD), and is a promising tool for assessing the response to the treatment of the disease. TARC also exists within platelets, with elevated levels detectable in AD patients. We examined the effects of pre-analytical factors on the quantitation of TARC in human EDTA plasma. TARC levels in platelet-free plasma were significantly lower than those in platelet-containing plasma. After freeze-thaw, TARC levels increased in platelet-containing plasma, but remained unchanged in platelet-free plasma, suggesting TARC was released from the platelets during the freeze-thaw process. In contrast, TARC levels were stable in serum independent of freeze-thaw. These findings underscore the importance of pre-analytical factors to TARC quantitation. Plasma TARC levels should be measured in platelet-free plasma for accurate quantitation. Pre-analytical factors influence the quantitation, interpretation, and implementation of circulating TARC as a biomarker for the development of AD therapeutics.

## 1. Introduction

The hallmark of inflammation is the infiltration of specific leukocyte subsets from the blood into the affected tissue [1]. Chemokines, which are small (8–10 kDa) secreted proteins and their receptors expressed on the cell surface, orchestrate the guided migration of leukocytes to inflammatory sites. Thymus- and activation-regulated chemokine (TARC/CCL17) is a high-affinity ligand for CC chemokine receptor 4 (CCR4), as well as being a Th2 type, pro-allergic secreted chemokine. TARC serves in the recruitment and migration of CCR4-expressing cells, such as Th2 cells, cutaneous leukocyte-associated antigen (CLA)^+^ skin-homing T cells, and CD25^+^ T suppressor cells [2]. It is closely related to the pathogenesis of allergic diseases with the accumulation of Th2 lymphocytes in the inflammatory regions. In inflamed skin lesions, TARC acts on CCR4 at the endothelial surface to promote vascular recognition for subsequent cellular localization [3]. TARC is constitutively expressed in the thymus [4], and is also present in monocyte-derived dendritic cells [5][Bibr bibr6-61749]–[7], endothelial cells [8], and epidermal keratinocytes [9].

Circulating levels of TARC have been associated with allergic skin diseases such as atopic dermatitis (AD), contact dermatitis, and psoriasis, in addition to asthma [10] and epilepsy [11], but not allergic respiratory diseases such as allergic rhinitis and allergic asthma [12][Bibr bibr13-61749]–[14]. Elevated levels of TARC have been reported in the serum of patients with AD, compared to that of healthy control subjects [12][Bibr bibr13-61749][Bibr bibr14-61749][Bibr bibr15-61749]–[16]. Similar observations have also been reported in the plasma of AD patients, compared to that of healthy control subjects [15, 16]. Moreover, the plasma or serum levels of TARC from AD patients correlated significantly with the severity of the disease, as classified by the objective SCORAD score [17, 18]. Thus, plasma/serum TARC is a disease activity marker for AD. Furthermore, plasma or serum levels of TARC from AD patients decreased after treatment, in accordance with the improvement of clinical symptoms [12][Bibr bibr13-61749][Bibr bibr14-61749]–[15, 19]. Therefore, plasma/serum TARC can also be utilized as a pharmacodynamic (PD) marker to assess disease pathway engagement in the clinical development of AD therapeutics.

In blood, TARC also exists within platelets. Platelets from AD patients contain higher levels of TARC than those found in healthy control subjects [15]. Consistent with the finding of the presence of TARC in platelets, serum TARC levels were much higher than those in plasma, owing to the release of TARC from the platelets during coagulation in serum preparation [15, 16]. Platelets may represent the predominant source of TARC in circulation, and platelet rupture, aggregation, or activation may affect levels of non-cell-associated TARC in blood. Thus, the alteration of platelets post-phlebotomy would artificially increase the levels of non-cell-associated TARC in blood. Although pre-analytical conditions may influence circulating TARC levels significantly, they have not been investigated thoroughly for the measurement of this marker.

In this article, we describe the effects of pre-analytical factors on TARC quantitation in human EDTA plasma, including the method for EDTA plasma preparation and plasma sample handling prior to sample analysis. Our observations underscore the importance of the influence of pre-analytical factors in clinical biomarker quantitation, interpretation, and implementation in drug development.

## 2. Material and Methods

### 2.1 Ethical conduct of research

The authors state that they have obtained appropriate institutional review board approval, or have followed the principles outlined in the Declaration of Helsinki, for all human or animal experimental investigations. In addition, for investigations involving human subjects, informed consent has been obtained from the participants involved.

### 2.2 Reagents

Recombinant human TARC protein was purchased from R&D Systems, Inc. (Minneapolis, MN, USA; Catalogue Number: 364-DN). The human TARC ultra-sensitive kit was purchased from Meso Scale Discovery (MSD) (Rockville, USA; Catalogue Number: K151BGC-2). The kit was supplied with cytokine panel 2 ultra-sensitive plate (Catalogue Number: N45016A-1); human TARC detection antibody (Catalogue Number: D21BG-3); cytokine panel 2 calibrator blend (Catalogue Number: C0016-2); Diluent 2 (Catalogue Number: R51BB-3); Diluent 3 (Catalogue Number: R51BA-5); and MSD Read Buffer T (4x) (Catalogue Number: R92TC-3). DPBS (Dulbecco's phosphate-buffered saline) (1x) was purchased from Mediatech, Inc. (Manassas, VA, USA; Catalogue Number: 21-031-CV). Surfact-Amps 20 (10% Tween 20) was purchased from Thermo Fisher Scientific (Rockford, IL, USA; Catalogue Number: CAS 9005-64-5). Blocker Casein in PBS (1% (w/v) casein) was also purchased from Thermo Fisher Scientific (Catalogue Number: 37528). HBR1 (heterophilic blocking reagent 1) was purchased from Scantibodies Laboratory, Inc. (Santee, CA, USA; Catalogue Number: 3KC534-075). StabilZyme SELECT stabilizer was purchased from SurModics, Inc. (Eden Prairie, MN, USA; Catalogue Number: SZ03-1000). Distilled water was purchased from Life Technologies (Grand Island, NY, USA; Catalogue Number: 15230-147).

EDTA plasma samples of healthy control subjects were purchased from Bioreclamation, Inc. (Hicksville, NY, USA).

### 2.3 Sample collection

Human plasma and serum samples from apparently healthy subjects were obtained with informed consent. For plasma preparation, whole blood was drawn into lavender top vacutainer tubes (6 mL, K_2_EDTA (spray dried), BD #367863). The blood tubes were mixed by gentle inversion 10x, incubated at room temperature (RT) for 60 min, and subsequently centrifuged at 1500 g for 15 min at RT, or prepared as described in Figure 3. EDTA plasma samples were collected, aliquoted, and stored at −80°C. For serum preparation, whole blood was drawn into red top vacutainer tubes (6 mL, clot-activator- and silicone-coated interior, BD #367815). The blood tubes were incubated at RT for 30 min, and then centrifuged at 1500 g for 15 min at RT. Serum samples were collected, aliquoted, and stored at −80°C.

### 2.4 Freeze-thaw treatment of plasma and serum samples

A freeze-thaw treatment of the plasma or serum samples was executed using the following procedure. Samples (100 μL per aliquot) were stored at −80°C for at least 4 h. They were then removed from the −80°C freezer and thawed at RT for 2 h. After one complete freeze-thaw cycle, samples were either analysed in the TARC assay or stored at −80°C.

### 2.5 Preparation of standards and QC samples

Recombinant human TARC protein (one vial, 25 μg) was reconstituted in 2.5 mL of StabilZyme SELECT stabilizer to a final concentration of 10 μg/mL. Then 200 μL of 10 μg/mL TARC was mixed with 199.8 mL of Casein-T (1% Casein and 0.05% Tween 20 in PBS) to obtain the TARC standard stock at 10, 000 pg/mL. The TARC standard stock was aliquoted and stored at −80°C. Prior to each analysis, standard samples were prepared by serial dilution of the TARC standard stock with Casein-T, resulting in a nine-point standard curve with a range of 1.5–10, 000 pg/mL.

Four sets of quality control (QC) samples (Extra High, High, Medium, and Low) were prepared by spiking recombinant human TARC protein into a human plasma sample purchased from Bioreclamation, Inc. The QC samples were aliquoted and stored at −80°C.

### 2.6 TARC ECL assay procedure and sample analysis

All samples (plasma, serum, standards, QC, and blank) were analysed manually in duplicate on an MSD-kit-supplied 96-well plate. All reagents were brought to room temperature prior to analysis. Plasma/serum samples and QC samples were diluted 1:4 in a [Casein-T + HBR] buffer, containing 0.4 mg/mL HBR1 in Casein-T (1.2 μg of HBR1 per μL of plasma or serum). A plate with 150 μL Casein per well was incubated for 1.5 h with vigorous shaking (1000 rpm) at RT. The plate was then washed three times with 200 μL per well of PBS-T (0.05% Tween 20 in PBS). After the addition of 50 μL Casein-T per well, and 50 μL of standards, diluted samples, or QC samples, the plate was incubated at RT for 2 h with vigorous shaking (1000 rpm). Upon completion of the sample incubation, the plate was washed with PBS-T buffer three times, and 25 μL of human TARC detection antibody were added to each well. The plate was incubated for 2 h with vigorous shaking (1000 rpm) at RT. The plate was again washed with PBS-T buffer three times. After the addition of 150 μL of 2x Read Buffer T (1:2 dilution of 4x Read Buffer T in distilled water) to each well, the plate was read on the MSD Sector Imager 6000.

### 2.7 Software and statistical analysis

The MSD DISCOVERY WORKBENCH® analysis software was used for data acquisition and data analysis. The standard curve was modelled using a least-squares fitting algorithm, meaning that signals from standards with known levels of TARC could be used to calculate the concentration of TARC in the sample. The software utilized a four-parameter logistic model and included a 1/Y2 weighting function to determine the mean, standard deviation (stdev), and % coefficient of variance (%CV). Back-calculated concentrations and % difference of the back-calculated concentrations were calculated using Microsoft Excel.

## 3. Results and Discussion

### 3.1 Differential effects of freeze-thaw on TARC quantitation in human EDTA plasma and serum

During the development of an ultra-sensitive electroche-miluminescence (ECL) assay for TARC quantitation in human EDTA plasma, we observed that increased levels of TARC were detected after repeated freeze-thaw cycles [20]. We decided to further examine the effects of freeze-thaw on TARC quantitation in EDTA plasma, and in serum as a comparison, including freshly prepared plasma and serum samples without any freeze-thaw. EDTA plasma and subject-matched serum samples were prepared simultaneously from six apparently healthy subjects following standard procedures, with low-speed centrifugation at 1500 g to separate blood cells from the plasma or serum. Aliquots of the freshly prepared plasma or serum samples were either analysed in the TARC assay immediately after sample preparation, or frozen and stored at −80°C. After the freeze-thaw treatment, the fresh frozen samples and fresh frozen samples following additional freeze-thaw cycles were also analysed in the TARC assay. A fresh frozen plasma/serum sample was defined as a plasma/serum sample that was frozen immediately after sample preparation. Therefore, every fresh frozen plasma/serum sample had already gone through one freeze-thaw cycle prior to the sample analysis for TARC measurement.

**Figure 1.**
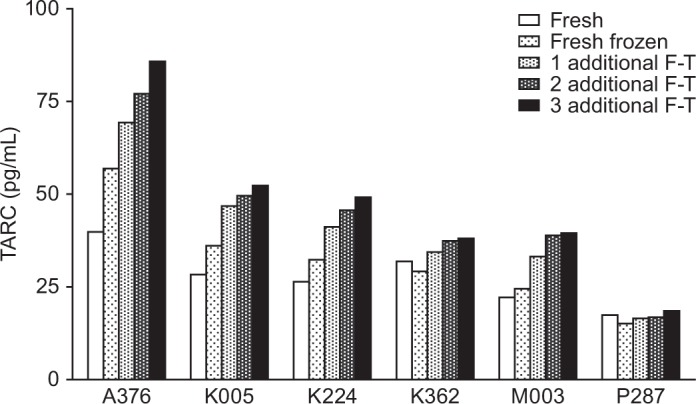
Effects of freeze-thaw (F-T) cycles on TARC levels in plasma. TARC levels were quantitated in EDTA plasma samples from six subjects, prepared by low-speed centrifugation (1500 g): fresh plasma, fresh frozen plasma, and fresh frozen plasma samples following one, two, or three additional F-T cycles.

Figure 1 illustrates the effects of freeze-thaw cycles on TARC quantitation in plasma samples. In general, the TARC levels in the fresh frozen plasma that had gone through one freeze-thaw cycle were higher than those in the fresh plasma without freeze-thaw. Exceptions were observed in certain subject samples, such as K362 and P287. In addition, the difference in TARC levels between the fresh and the fresh frozen plasma was particularly large in some subjects, such as subject A376. Consistent with our previous observations, compared to the fresh frozen plasma, TARC levels increased in plasma that had gone through one additional freeze-thaw cycle for all subjects. Furthermore, increased levels of TARC were detected in plasma with repeated freeze-thaw cycles, up to at least three additional cycles.

**Figure 2.**
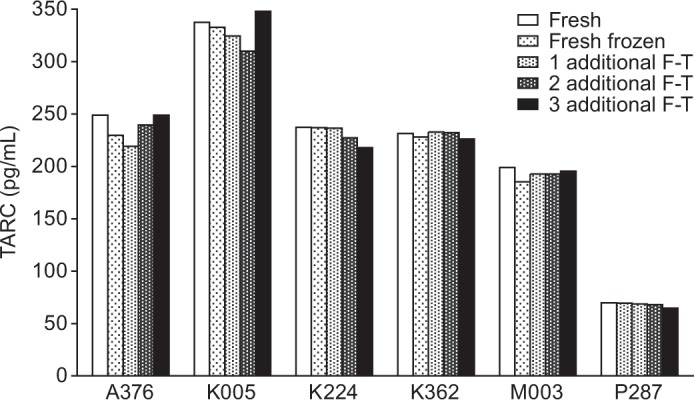
Effects of freeze-thaw (F-T) cycles on TARC levels in serum. TARC levels were quantitated in serum samples from six subjects, prepared by low-speed centrifugation (1500 g): fresh serum, fresh frozen serum, and fresh frozen serum samples following one, two, or three additional F-T cycles. The blood draws for the serum and the EDTA plasma (Figure 1) were performed during the same visit.

In contrast to the observations in plasma, in subject-matched serum, TARC levels remained unchanged between the fresh and the fresh frozen samples for all subjects, even after three additional freeze-thaw cycles (Figure 2). The blood draws for serum and subject-matched EDTA plasma were performed during the same visit, and the serum and EDTA plasma samples were prepared at the same time. Consistent with previous observations, TARC levels in serum were much higher than those in plasma, probably owing to the release of TARC from platelets during coagulation (30 min whole blood incubation at RT) in serum preparation (compare Figure 1 and Figure 2) [15, 16]. In addition, the comparable levels of TARC detected in fresh serum and serum that had gone through multiple freeze-thaw cycles suggested that almost all TARC in the platelets was released into serum during coagulation; therefore, TARC levels in fresh serum were much higher than those in fresh plasma. The large ratio of TARC levels in serum versus plasma — 4–12 fold in six subjects —suggested that platelets are the predominant source of circulating TARC.

### 3.2 TARC levels in platelet-free, but not platelet-containing, EDTA plasma were independent of freeze-thaw

We were intrigued by the differential effects of freeze-thaw on TARC quantitation in matched EDTA plasma and serum samples from six individual donors (Figure 1 and Figure 2). The fact that freeze-thaw induced increased TARC levels in plasma, but not in serum, led us to hypothesize that plasma samples may have contained residual platelets, and that these platelets released TARC during freeze-thaw and contributed to the increase of TARC in plasma. The EDTA plasma samples used in these experiments were prepared following the guidelines of a standard plasma preparation method (National Cancer Institute, The Early Detection Research Network (EDRN) Standard Operating Procedure (SOP) for Collection of EDTA Plasma, http://edrn.nci.nih.gov/resources/standard-operating-procedures/standard-operating-procedures/biological-specimens), with low-speed centrifugation of whole blood at 1500 g for 15 min. These plasma samples most likely contained residual platelet cells from the whole blood. We then investigated the effect of the whole blood centrifugation speed on TARC levels in plasma. In particular, we examined TARC levels in platelet-containing and platelet-free EDTA plasma samples.

**Figure 3.**
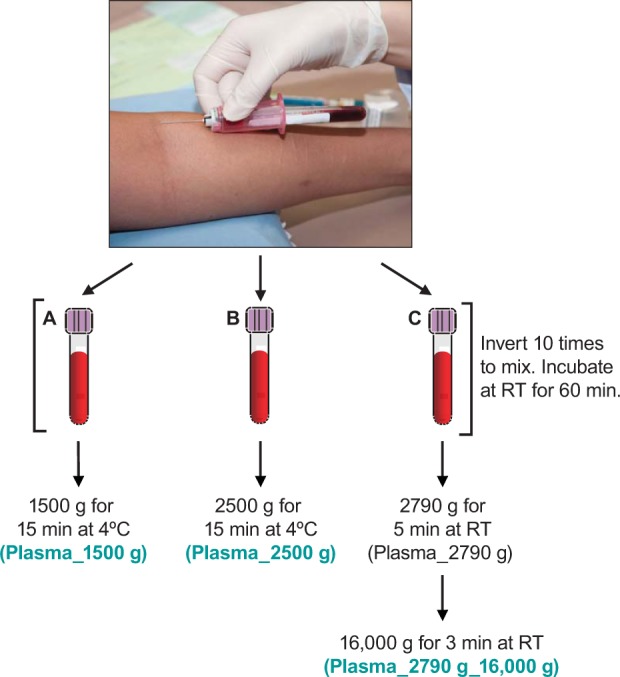
Preparation of platelet-containing and platelet-free EDTA plasma samples. Venous blood was collected into three purple/lavender top EDTA vacutainer tubes. After the blood tubes were inverted 10 times to mix thoroughly, and incubated at room temperature (RT) for 60 min, EDTA plasma samples were prepared following three methods: (A) centrifugation of the blood tube at 1500 g for 15 min at 4°C, to generate platelet-containing EDTA plasma; (B) centrifugation of the blood tube at 2500 g for 15 min at 4°C, to generate platelet-free EDTA plasma [22]; and (C) centrifugation of the blood tube at 2790 g for 5 min at RT to separate cells from the plasma, then re-centrifugation of the plasma at 16, 000 g for 3 min at RT to remove any residual platelets, for platelet-free EDTA plasma [23].

Varo et al. performed a thorough analysis of the preparation of platelet-rich and platelet-free plasma, by increasing the speed of whole blood centrifugation (which gradually depletes platelets and leucocytes from plasma), and discovered that at least 2000 g centrifugal force is required to produce platelet-free plasma samples [21]. Thus, plasma samples prepared through centrifugation at 1500 g most likely contain residual platelet cells. In addition, Ivandic et al. [22] and Halldorsdottir et al. [23] have described two platelet-free plasma preparation methods, involving whole blood centrifugation greater than 2000 g. Centrifugation at high speed, as described in these two methods, did not shear platelet cells. Here, we prepared EDTA plasma using three different methods (Figure 3): the standard low-speed centrifugation method (1500 g), which produces platelet-containing EDTA plasma (method A), as well as two additional methods described in the literature, which allow the preparation of platelet-free EDTA plasma (methods B and C) [22, 23]. The major difference between these three methods is centrifugation speed, which determines whether platelet cells in whole blood are completely separated from EDTA plasma or not. Additional variables in EDTA plasma preparation (such as the use of glass versus plastic collection tubes) could potentially impact the success of an assay [23, 24]. Nevertheless, the scope of this study is to compare the effects of platelet-containing and platelet-free EDTA plasma in TARC quantitation.

From each of the six apparently healthy subjects, we collected three tubes of venous blood and prepared three different types of EDTA plasma samples, following the methods illustrated in Figure 3: (A) Plasma_1500 g, (B) Plasma_2500 g, and (C) Plasma_2700 g_16, 000 g. The fresh frozen plasma samples and the fresh frozen plasma samples following additional freeze-thaw cycles were analysed in the TARC assay. Three subjects — A376, K362, and M003 — participated in this study in addition to the study of freeze-thaw effects on TARC levels in plasma and matched serum samples described above. However, the blood draws for these two studies were five weeks apart. Thus, the plasma TARC levels of the same subject in these two studies cannot be compared directly, owing to intra-subject biological variability.

**Figure 4.**
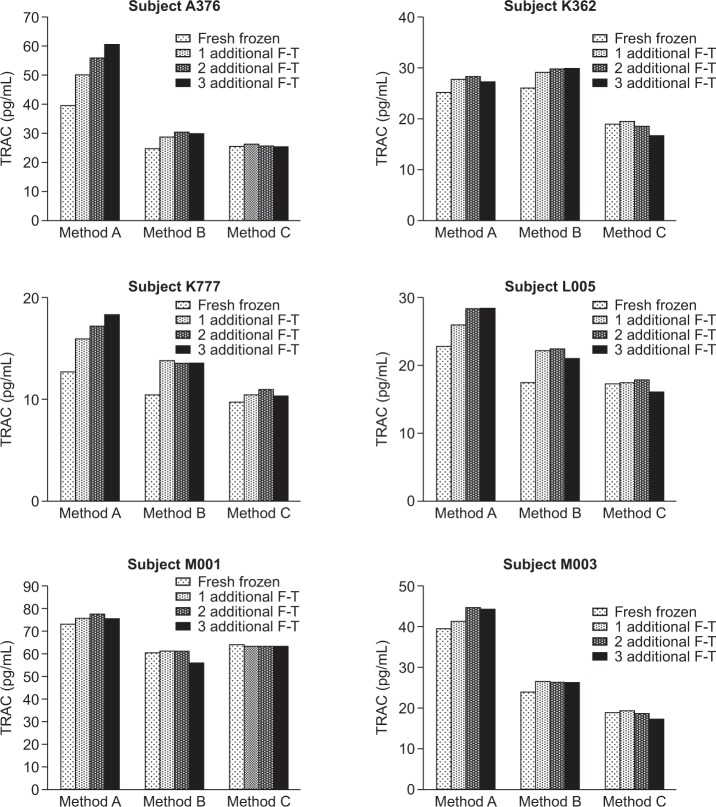
Differential effects of three different EDTA plasma preparation methods on TARC quantitation in plasma. Plasma samples were prepared by method A, B, or C, as illustrated in Figure 3, from six apparently healthy subjects: A376, K362, K777, L005, M001, and M003. The TARC levels in the fresh frozen plasma and in the plasma samples that went through an additional one, two, or three freeze-thaw cycles were quantified.

Figure 4 illustrates the differential effects of three different EDTA plasma preparation methods on TARC levels from six apparently healthy subjects. For each plasma preparation method, TARC levels in the fresh frozen plasma and the plasma samples following three additional freeze-thaw cycles were quantified. Again, in the platelet-containing EDTA plasma samples (Plasma_1500 g), compared to the fresh frozen plasma, elevated levels of TARC were observed in the plasma following repeated freeze-thaw cycles, for all six subjects. In contrast, TARC levels remained unchanged (<20% difference) even after at least three additional freeze-thaw cycles, in platelet-free EDTA plasma prepared following method C (Plasma_2700 g_16, 000 g). After one additional freeze-thaw cycle, the difference between TARC levels in method A (Plasma_1500 g) and method C (Plasma_2700 g_16, 000 g) was significant between the six subjects, with a p value of 0.008 determined by a paired T test. Furthermore, this significant difference was maintained with an additional second and third freeze-thaw cycle, with p values of 0.009 and 0.011, respectively. In the platelet-free EDTA plasma prepared following method B (Plasma_2500 g), TARC levels increased after one additional freeze-thaw cycle. These observations suggest that there was sufficient platelet contamination in the EDTA plasma prepared by low-speed centrifugation, and that these platelets released TARC during freeze-thaw, leading to elevated TARC levels in the plasma after repeated freeze-thaw cycles. In addition, it is possible that platelet activation could also contributed to the increased TARC levels in the EDTA plasma prepared by low-speed centrifugation although it is unlikely. Therefore, it was platelet contamination (and perhaps activation) that caused the TARC accumulation in the stored frozen plasma.

Furthermore, TARC levels were consistently lower in samples that underwent high-speed centrifugation in all six subjects (Figure 4: compare the TARC levels in plasma prepared by method A and method C). We hypothesize that this was owing to the elimination of residual platelets achieved with the 16, 000 g centrifugal force. We recognize that it is also possible that a higher centrifugal force may minimize the activation of platelets, and thus the release of TARC. Similar findings have previously been reported; for instance, Morita et al. reported that, in EDTA plasma samples prepared by whole blood centrifugation at 2000 g, TARC levels were not altered after at least five freeze-thaw cycles [16], suggesting that there was no or minimal platelet contamination in these plasma samples. Data from the freeze-thaw study, however, was not shown in the report [16], making it difficult to perform a careful comparison with the results that we observed (Figure 4). Fujisawa et al. reported TARC levels in EDTA plasma samples prepared by whole blood centrifugation at 1800 g [15], but unfortunately the effect of freeze-thaw cycles on the TARC levels in these plasma samples was not examined.

The use of TARC in clinical studies requires reliable sample preparation and analytical measurement. Our present results underscore the importance of the careful removal of platelets to determine free TARC in blood and to avoid interference with the ligand derived from the cell-associated pools. Plasma prepared in this manner can undergo at least three freeze-thaw cycles without perturbing the analysis. It is very important to avoid platelet contamination in plasma preparation because variabilities in platelet abundance, TARC expression in platelet, level of residual platelets in plasma, and degree of ruptured platelets in plasma after freeze-thaw can lead to incorrect interpretation of TARC in plasma, the biomarker data in a clinical study. The assessment of citrated plasma or plasma collected in CTAD (citrate, theophylline, adenosine, and dipyridamole) tubes for TARC quantitation is warranted. Both of these tube types are known to help prevent ex-vivo platelet activation [22]. Platelets produce a number of pro-inflammatory mediators during activation; as with TARC, circulating levels of these mediators are complicated in platelet-containing plasma. These mediators include vascular endothelial growth factor (VGEF), which is a platelet-derived growth factor involved in both angiogenesis and pulmonary capillary permeability [25]; platelet factor 4 (PF4), which is a cytokine stored in platelets and released during activation [25]; and soluble CD40 ligand (sCD40L), which is a trans-membrane protein released from the platelet in its soluble form during platelet activation, and is involved in inflammation and thrombosis [21][Bibr bibr22-61749]–[23, 25]. Thus, investigators should be cautious of the choice of specimen and specimen-processing method when measuring circulating biomarkers that also exists in platelets.

## 4. Conclusions

In this article, we have presented the influence of pre-analytical factors in the quantitation of circulating TARC levels. Owing to its presence in platelets, the circulating levels of this pro-inflammatory chemokine were dramatically altered in platelet-containing EDTA plasma samples and were further complicated after repeated freeze-thaw cycles. Therefore, the choice of specimen type and specimen-handling procedures should be carefully considered for the measurement of circulating non-cell-associated TARC. When using TARC as a biomarker to aid the clinical development of atopic dermatitis therapeutics, the optimum strategy would be to measure TARC in platelet-free plasma or serum.

## 6. Conflict of Interest

X. Zhao and O. F. Laterza are employees of Merck Sharp & Dohme. L. Delgado and R. Weiner were employees of Merck Sharp & Dohme during the course of this work. X. Zhao and O. F. Laterza own company stock/stock options. The authors have no other relevant affiliations or financial involvement with any organization or entity with a financial interest in or financial conflict with the subject matter or materials discussed in the manuscript apart from those disclosed.

No writing assistance was utilized in the production of this manuscript.

## References

[1] Ben-Baruch A Michiel DF Oppenheim JJ. Signals and receptors involved in recruitment of inflammatory cells. J Biol Chem. 1995 May 19;270:11703–6. 7744810 10.1074/jbc.270.20.11703

[2] Zlotnik A Yoshie O. The chemokine superfamily revisited. Immunity. 2012 May 25;36:705–16. 22633458 10.1016/j.immuni.2012.05.008 PMC3396424

[3] Mariani M Lang R Binda E Panina-Bordignon P D'Ambrosio D. Dominance of CCL22 over CCL17 in induction of chemokine receptor CCR4 desensitization and internalization on human Th2 cells. Eur J Immunol. 2004 Jan;34:231–40. 14971049 10.1002/eji.200324429

[4] Imai T Yoshida T Baba M Nishimura M Kakizaki M Yoshie O. Molecular cloning of a novel T cell-directed CC chemokine expressed in thymus by signal sequence trap using Epstein-Barr virus vector. J Biol Chem. 1996 Aug 30;271:21514–21. 8702936 10.1074/jbc.271.35.21514

[5] Hashimoto S Suzuki T Dong HY Nagai S Yamazaki N Matsushima K. Serial analysis of gene expression in human monocyte-derived dendritic cells. Blood. 1999 Aug 1;94:845–52. 10419874

[bibr6-61749] Sallusto F Schaerli P Loetscher P Schaniel C Lenig D Mackay CR Rapid and coordinated switch in chemokine receptor expression during dendritic cell maturation. Eur J Immunol. 1998 Sep;28:2760–9. 9754563 10.1002/(SICI)1521-4141(199809)28:09<2760::AID-IMMU2760>3.0.CO;2-N

[7] Sallusto F Palermo B Lenig D Miettinen M Matikainen S Julkunen I Distinct patterns and kinetics of chemokine production regulate dendritic cell function. Eur J Immunol. 1999 May;29:1617–25. 10359116 10.1002/(SICI)1521-4141(199905)29:05<1617::AID-IMMU1617>3.0.CO;2-3

[8] Campbell JJ Haraldsen G Pan J Rottman J Qin S Ponath P The chemokine receptor CCR4 in vascular recognition by cutaneous but not intestinal memory T cells. Nature. 1999 Aug 19;400:776–80. 10466728 10.1038/23495

[9] Vestergaard C Yoneyama H Murai M Nakamura K Tamaki K Terashima Y Overproduction of Th2-specific chemokines in NC/Nga mice exhibiting atopic dermatitis-like lesions. J Clin Invest. 1999 Oct;104:1097–105. 10525048 10.1172/JCI7613 PMC408579

[10] Leung TF Wong CK Lam CW Li AM Ip WK Wong GW Plasma TARC concentration may be a useful marker for asthmatic exacerbation in children. Eur Respir J. 2003 Apr;21:616–20. 12762345 10.1183/09031936.03.00083303

[11] Pollard JR Eidelman O Mueller GP Dalgard CL Crino PB Anderson CT The TARC/sICAM5 Ratio in Patient Plasma is a Candidate Biomarker for Drug Resistant Epilepsy. Front Neurol. 2012;3:181. 23293627 10.3389/fneur.2012.00181 PMC3535822

[12] Hijnen D De Bruin-Weller M Oosting B Lebre C De Jong E Bruijnzeel-Koomen C Serum thymus and activation-regulated chemokine (TARC) and cutaneous T cell- attracting chemokine (CTACK) levels in allergic diseases: TARC and CTACK are disease-specific markers for atopic dermatitis. J Allergy Clin Immunol. 2004 Feb; 113:334–40. 14767451 10.1016/j.jaci.2003.12.007

[bibr13-61749] Kakinuma T Nakamura K Wakugawa M Mitsui H Tada Y Saeki H Thymus and activation-regulated chemokine in atopic dermatitis: Serum thymus and activation-regulated chemokine level is closely related with disease activity. J Allergy Clin Immunol. 2001 Mar;107:535–41. 11240957 10.1067/mai.2001.113237

[bibr14-61749] Shimada Y Takehara K Sato S. Both Th2 and Th1 chemokines (TARC/CCL17, MDC/CCL22, and Mig/CXCL9) are elevated in sera from patients with atopic dermatitis. J Dermatol Sci. 2004 May;34:201–8. 15113590 10.1016/j.jdermsci.2004.01.001

[bibr15-61749] Fujisawa T Fujisawa R Kato Y Nakayama T Morita A Katsumata H Presence of high contents of thymus and activation-regulated chemokine in platelets and elevated plasma levels of thymus and activation-regulated chemokine and macrophage-derived chemokine in patients with atopic dermatitis. J Allergy Clin Immunol. 2002 Jul; 110:139–46. 12110833 10.1067/mai.2002.126079

[16] Morita A Kikuoka S Horikawa T Bito T Yamada H Kanda M Evaluation of human thymus and activation-regulated chemokine concentrations in blood using a new sandwich ELISA based on monoclonal antibodies. Clin Chim Acta. 2002 Aug; 322:67–75. 12104083 10.1016/s0009-8981(02)00131-6

[17] Severity scoring of atopic dermatitis: the SCORAD index. Consensus Report of the European Task Force on Atopic Dermatitis. Dermatology. 1993;186:23–31. 8435513 10.1159/000247298

[18] Kunz B Oranje AP Labrèze L Stalder JF Ring J Taïeb A. Clinical validation and guidelines for the SCORAD index: consensus report of the European Task Force on Atopic Dermatitis. Dermatology. 1997;195:10–9. 10.1159/000245677 9267730

[19] Haeck IM Knol MJ ten Berge O van Velsen SG de Bruin-Weller MS Bruijnzeel-Koomen CA. Enteric-coated mycophenolate sodium versus cyclosporin A as long-term treatment in adult patients with severe atopic dermatitis: a randomized controlled trial. J Am Acad Dermatol. 2011 Jun;64:1074–84. 21458107 10.1016/j.jaad.2010.04.027

[20] Zhao X Delgado L Weiner R Laterza OF. An ultra-sensitive clinical biomarker assay: quantitation of thymus and activation-regulated chemokine in human plasma. Bioanalysis. 2014 Apr;6:1069–80. 24830891 10.4155/bio.14.72

[21] Varo N Nuzzo R Natal C Libby P Schönbeck U. Influence of pre-analytical and analytical factors on soluble CD40L measurements. Clin Sci (Lond). 2006 Nov;111:341–7. 16856875 10.1042/CS20060047

[bibr22-61749] Ivandic BT Spanuth E Haase D Lestin HG Katus HA. Increased plasma concentrations of soluble CD40 ligand in acute coronary syndrome depend on in vitro platelet activation. Clin Chem. 2007 Jul; 53:1231–4. 17495016 10.1373/clinchem.2007.085332

[23] Halldórsdóttir AM Stoker J Porche-Sorbet R Eby CS. Soluble CD40 ligand measurement inaccuracies attributable to specimen type, processing time, and ELISA method. Clin Chem. 2005 Jun;51:1054–7. 15914794 10.1373/clinchem.2005.048199

[24] Tuck MK Chan DW Chia D Godwin AK Grizzle WE Krueger KE Standard operating procedures for serum and plasma collection: early detection research network consensus statement standard operating procedure integration working group. J Proteome Res. 2009 Jan;8:113–7. 19072545 10.1021/pr800545q PMC2655764

[25] Bercovitz RS Kelher MR Khan SY Land KJ Berry TH Silliman CC. The pro-inflammatory effects of platelet contamination in plasma and mitigation strategies for avoidance. Vox Sang. 2012 May; 102:345–53. 22092073 10.1111/j.1423-0410.2011.01559.x PMC3690768

